# Unilateral amelia with limb deformities and multiple congenital malformations in a newborn: a case report from Palestine

**DOI:** 10.1097/MS9.0000000000002913

**Published:** 2025-02-11

**Authors:** Majd Oweidat, Mohammed Alra’e, Mohammed Aldwaik, Abdalhakim Shubietah

**Affiliations:** a Department of Surgery, College of Medicine, Hebron University, Hebron, Palestine; b Department of Pediatrics, College of Medicine, Hebron University, Hebron, Palestine; c Department of Pediatrics, Princess Alia Governmental Hospital, Hebron, Palestine; d Department of Internal Medicine, Advocate Illinois Masonic Medical Center, Chicago, Illinois, USA

**Keywords:** amelia, case report, congenital limb defects, genetic syndromes, intercalary aplasia

## Abstract

**Introduction:**

Unilateral amelia, a rare congenital anomaly characterized by the absence of one limb, is often accompanied by severe malformations in other systems. Although the survival of affected infants beyond the neonatal period is rare, some cases have been documented with varying outcomes. This case report discusses a newborn with unilateral amelia and multiple congenital anomalies.

**Presentation of case:**

A 41-week gestation stillbirth from Palestine was delivered after a normal pregnancy, except for prenatal ultrasound findings of absent left upper limb, severe lower limb deformities, preaxial polydactyly, syndactyly, dextrocardia, and asymmetric hydrocephalus. Postnatal examination revealed additional craniofacial anomalies, gastrointestinal malformations, and respiratory abnormalities. Despite the severe deformities, the infant survived beyond 2 months.

**Discussion:**

Unilateral amelia is often associated with other congenital malformations, indicating complex embryological disruptions. The combination of limb deficiency with visceral abnormalities complicates management and prognosis. While stillbirths and early neonatal deaths are common, this case’s survival beyond 2 months is a notable exception. This report contributes valuable insight into the prognosis of such rare congenital conditions.

**Conclusion:**

This case emphasizes the rarity of unilateral amelia with multiple congenital defects and underscores the importance of comprehensive prenatal evaluation and genetic counseling. The patient’s extended survival provides new perspectives on the management and outcomes of infants with such complex anomalies.

## Introduction

Amelia is defined as the unilateral or bilateral complete absence of one or more upper or lower limbs with varying degrees of severity depending on the extent of embryogenesis disruption. Amelia can occur as an isolated defect or as part of a genetic syndrome, resulting from a longitudinal failure in limb formation, specifically the absence of an intervening segment of the extremity (intercalary aplasia)^[1]^.

**Highlights**

- Unilateral amelia with multiple congenital malformations in a male neonate.
- Absence of left upper limb and radial ray defect in right upper limb detected.
- Dextrocardia with multiple cardiac defects, ventriculomegaly, and scoliosis.
- Early prenatal ultrasonography played a key role in diagnosis.
- Genetic testing unavailable due to financial limitations in resource-limited settings.

The overall prevalence of amelia is 2.43 per 100 000 births, with a prevalence among live births of 0.63 per 100 000; of these cases, 70% involve the lower limbs, and the condition is associated with a high infant mortality rate of 67%. There is no significant difference in the frequency of limb deficiencies between the left and right sides of the body^[2]^.

Amelia can result from various etiologies, including thalidomide exposure, alcohol use, genetic factors, and maternal conditions such as diabetes mellitus. On the contrary, taking multivitamins during the periconceptional period may help reduce the risk of limb defects^[3,4]^.

Studies have shown that amelia is associated with various defects, including musculoskeletal malformations, spinal anomalies, large intestine atresia, renal agenesis, indeterminate sex, congenital foot deformities, cleft palate with cleft lip, and cardiac septal defects^[5]^.

To showcase the rarity of this congenital anomaly, we report a unique case of amelia with findings of absence of the left upper limb, a radial ray defect in the right upper limb with a missing radius, carpal bone abnormalities, and an absent thumb. Additionally, the left foot exhibited a split foot deformity, preaxial polydactyly, and syndactyly in a newborn patient at our institution. The male newborn also had several cardiac, neurologic, and musculoskeletal abnormalities.

Our work has been reported in line with the CAse REport guidelines 2017 criteria^[6]^.

## Presentation of case

We report a male neonate born following a non-consanguineous conception. The mother is in her early 20s, Gravida 2, Para 1, Abortus 1, while the father is in his late 20s, and both are healthy. The pregnancy was closely monitored with routine antenatal care. There was no history of teratogen exposure, alcohol consumption, or drug use during pregnancy. The mother experienced flu-like symptoms during the fourth month of gestation, which resolved spontaneously. The family history is unremarkable for congenital musculoskeletal defects or other anomalies.

The neonate was delivered at 37 weeks of gestation via normal vaginal delivery, with a birth weight of 2400 g, a height of 48 cm, and a head circumference of 33 cm. The anterior fontanelle was flat and of normal size. The infant is currently alive at 2 months of age.

**Figure 1.**
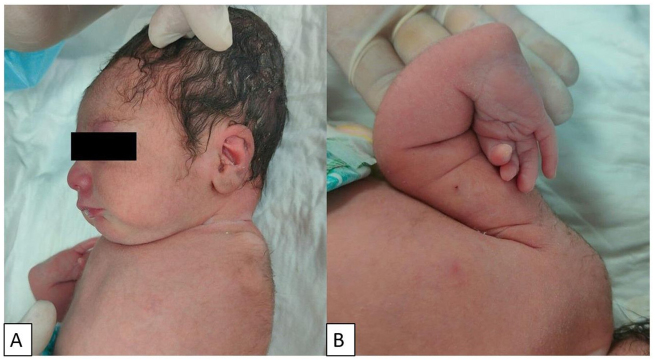
Two images show the amelia and the patient’s upper extremity deformities. (A) The absence of the upper limb (amelia) on the left side. The visible structure represents the bony prominence of the scapula without an attached stump. Caput succedaneum developed as a complication of vaginal delivery. (B) A radial ray defect on the right side with an absent thumb.

**Figure 2.**
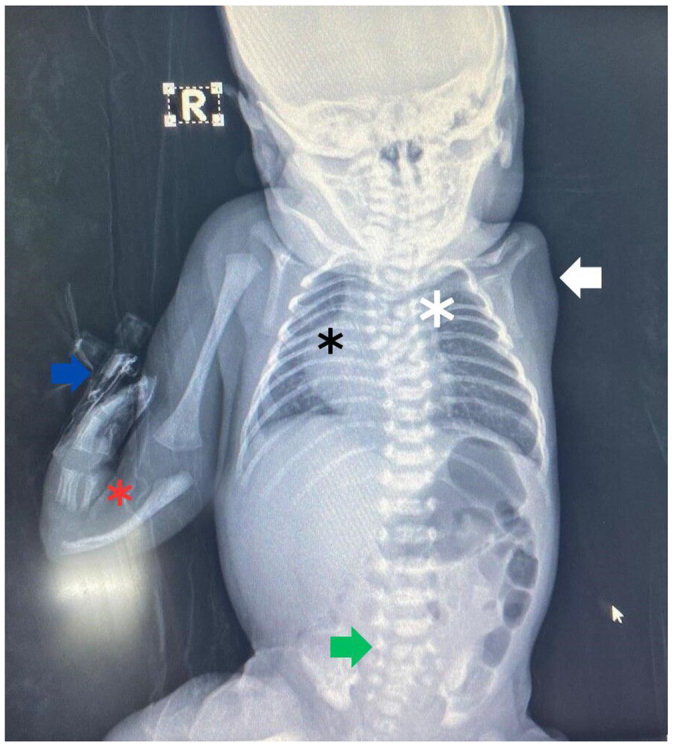
A babygram illustrates the radial ray defect on the right side, with the absence of both the radius (red asterisk) and the thumb. A cannula is connected to the dorsum of the right hand (blue arrow). Amelia is present on the left side, along with the absence of the bony portion of the humerus (white arrow). Additionally, the ribs on the right side are crowded (black asterisk). There are sagittal cleft vertebrae (butterfly vertebrae) in the upper dorsal spine (white asterisk) and in the first sacral vertebra (green arrow), with mild scoliosis of the upper dorsal spine, convex toward the right. The pelvic bones appear normal. The patient also has dextrocardia.

**Figure 3.**
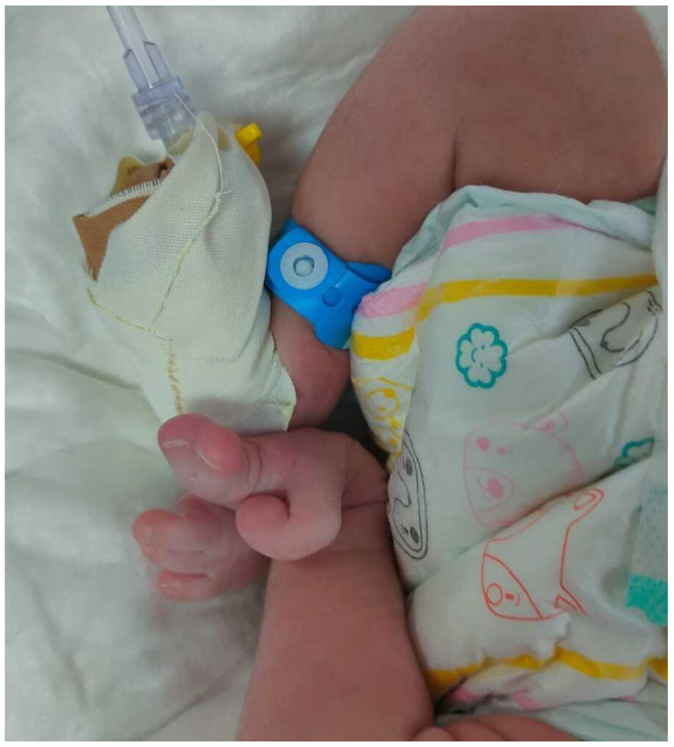
A normal right foot, and a preaxial polydactyly, syndactyly, and a split foot deformity on the left foot, from medial to lateral.

On examination, it was noted that the left upper limb was absent (Fig. 1A). The right upper limb exhibited a radial ray defect, characterized by the absence of the radius, abnormalities in the carpal bones, and an absent thumb (Figs 1B and 2). The left foot displayed a split foot deformity, along with preaxial polydactyly and syndactyly (Fig. 3), while the right foot appeared normal.

A facial examination revealed a depressed nasal bridge and micrognathia. Primitive reflexes were weak. The left testis was palpable within the scrotum, while the right testis was non-palpable within the scrotum or inguinal canal. The genitalia were consistent with normal male anatomy, and the anus was normally presented and positioned.

A grade 3 pansystolic murmur was auscultated on the right side of the anterior chest, raising suspicion of dextrocardia, which was subsequently confirmed by echocardiography and chest radiography (Fig. 2).

A detailed ultrasonography scan (DUSS) performed at 27 weeks of gestation revealed the complete absence of the left upper limb and the absence of the radius in the right upper limb. The right hand exhibited a wrist-drop appearance, and the left foot resembled a clubfoot deformity. Cardiac findings included a persistent left superior vena cava (PLSVC) and a dilated coronary sinus. The brain scan showed bilateral severe ventriculomegaly, a dilated third ventricle, and a dangling choroid plexus.

**Figure 4.**
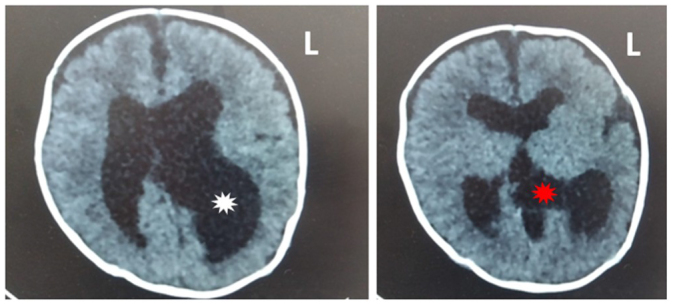
A brain CT scan showing asymmetric hydrocephalus. The left image highlights a more pronounced dilation of the left lateral ventricle (white asterisk) than the right. The right image demonstrates a dilated third ventricle (red asterisk). CT, computed tomography.

Postnatal ultrasonography of the internal organs indicated that the liver, gallbladder, spleen, kidneys, and urinary bladder were normal. A non-contrast computed tomography (CT) scan of the brain was performed, confirmed a dilated third ventricle, a normal fourth ventricle, and moderate asymmetric hydrocephalus, more pronounced in the left lateral ventricle (Fig. 4). The scan also revealed the absence of the septum pellucidum and a suspected dysgenesis of the corpus callosum.

Echocardiography findings were consistent with dextrocardia with situs solitus, moderate patent foramen ovale, moderate perimembranous ventricular septal defect (VSD), and PLSVC draining into a dilated coronary sinus. The right pulmonary artery was dilated, and the left pulmonary artery was stenosed.

A thoracoabdominal radiograph was performed (Fig. 2). which further revealed crowding of the ribs on the right side, sagittal cleft vertebrae (butterfly vertebrae) in the upper dorsal spine and the first sacral vertebra, and mild scoliosis of the upper dorsal spine with convexity to the right side. The pelvic bones were found to be normal.

The initial diagnostic workup for this patient revealed normal results for the complete blood count and basic metabolic panel. Additionally, the Toxoplasmosis, Rubella Cytomegalovirus, Herpes Simplex, and HIV panel was negative, effectively ruling out congenital infections. Neurological evaluation indicated the need for ongoing assessment by a pediatric neurologist, with serial imaging and follow-up for ventriculomegaly. The family was counseled on the signs of increased intracranial pressure, such as rapid head growth, vomiting, and irritability. Regular monitoring of developmental milestones was also emphasized as part of the care plan. Regarding the cardiac anomalies, the patient’s heart defects necessitate close follow-up with serial echocardiograms under the supervision of a pediatric cardiologist. At the time of evaluation, the defects did not cause hemodynamic instability, intracardiac, or extracardiac complications. Medical management included close monitoring of cardiac function and growth, along with prophylactic measures such as diuretics, angiotensin-converting enzyme inhibitors, and antibiotics, to prevent complications such as heart failure or infective endocarditis. The patient’s musculoskeletal deformities require intervention by an orthopedic specialist, with plans for reconstructive surgeries and prosthetic fittings to enhance functionality and quality of life. However, these interventions can be postponed until the child reaches an appropriate age. Lastly, comprehensive, long-term follow-up at a multidisciplinary center is strongly recommended to address the complex nature of the patient’s condition.

Although genetic testing was recommended to investigate the underlying etiology of the congenital malformations, the patient’s family was unable to pursue this option due to financial constraints.

## Discussion

This report describes a newborn with left upper limb amelia, radial ray defect, including absence of both radius and thumb of the right upper limb, and a combination of split foot deformity, preaxial polydactyly, and syndactyly of the left foot. Other findings include a depressed nasal bridge, micrognathia, dextrocardia, multiple congenital heart defects (PFO, VSD, PLSVC, and left pulmonary artery stenosis), dilated third and lateral ventricles in the brain, sagittal cleft vertebrae, and scoliosis of the upper spine, which present in up to 50% of patients with unilateral amelia^[7]^. The rarity of this case presentation prompted us to report it.

This case has an unremarkable family and pregnancy history. According to the patient’s mother, there were no teratogenic exposures or diabetes during the pregnancy. Both primiparity and young maternal age (<25 years) are associated with an increased risk of congenital limb deficiencies among all cases^[3,4]^, and both these risk factors are presented in our case.

Additionally, the presence of flu-like symptoms experienced by the mother during the fourth month of gestation raises suspicion about the correlation of infection with this case. Amelia was traditionally thought to be a sporadic anomaly with little risk of recurrence, or evidence of genetic origins. However, the autosomal dominant, autosomal recessive, and X-linked dominant modes of inheritance, have all been implicated in the possible etiology of amelia, which indicates the genetic heterogeneity of this condition^[8,9]^.

The presence of these clinical findings suggests a genetic syndrome. Vertebral anomalies, anal atresia, cardiac defects, tracheoesophageal fistula, esophageal atresia, renal anomalies, and limb defects (VACTERL) association is the most common genetic disorder associated with congenital limb defects^[8]^. It is defined by the presence of at least three of the following congenital malformations: vertebral defects, anal atresia, cardiac defects, tracheoesophageal fistula, renal anomalies, and limb abnormalities. Importantly, there should be no clinical or laboratory-based evidence for the presence of one of the many similar conditions, as the differential diagnosis is relatively large^[10]^. The findings in our patient are compatible with the VACTERL association, which is a diagnosis of exclusion that can be confirmed by ruling out other genetic causes.

Regarding limb defects and multiple malformations, Robert syndrome is a rare autosomal recessive genetic disorder characterized by prenatal and postnatal growth retardation, limb defects, and craniofacial anomalies^[11]^. The absence of craniofacial abnormalities in our patient makes this syndrome unlikely.

Additionally, this case presentation closely resembles Holt-Oram syndrome, which is caused by autosomal dominant disorder. This condition affects limb development, and it is often associated with cardiac anomalies like atrial septal defect or VSD^[12]^. While the clinical presentation of our patient aligns with many features of Holt-Oram syndrome, genetic testing is required to confirm the diagnosis due to the syndrome’s genetic heterogeneity and variable expressivity.

Unfortunately, genetic testing could not be performed in this case due to financial constraints faced by the family, highlighting a common limitation in resource-limited settings that impedes definitive diagnosis and future risk assessments. This underscores the need for greater access to genetic testing and counseling in similar cases, which could guide patient care and family planning.

Infants with isolated amelia appear to have a good prognosis, whereas the prognosis is poor in newborns with amelia, which is associated with organ malformations, as most of them die in the first year of life^[13]^. Our patient had healthy neonatal outcomes and is currently alive at 2 months of age, whereas most similar cases are either stillborn or die in their early neonatal period^[14,15]^.

Recent studies highlight various presentations of amelia with associated congenital anomalies. One case involved a 22-year-old primigravida whose fetus was diagnosed with absence of both upper and lower limbs and mild renal abnormalities; the baby was stillborn at 41 weeks despite counseling for poor prognosis^[16]^. Another case described a stillborn infant with complete limb absence, cleft lip, pulmonary agenesis, and cardiovascular malformations, underscoring the severity of associated organ defects^[17]^. A live neonate with absent limbs, cleft lip, and significant urological and spinal abnormalities also had a stable postnatal course and was referred for further management after 35 days^[18]^. Additionally, a study on scoliosis in patients with unilateral amelia reported that such cases may develop significant spinal deformities, requiring long-term interventions like growing rods^[19]^. These cases reinforce the variable presentation and complexity of amelia, emphasizing the importance of early diagnosis and multidisciplinary care. These cases reinforce the variable presentation and complexity of amelia, emphasizing the importance of early diagnosis and multidisciplinary care. However, the literature lacks many cases directly pertinent to the case report presented here, highlighting the rarity of this condition and the need for further research to understand its full spectrum.

Recurrent amelia has been documented in only a few families, making recurrence unlikely, as the family history was unremarkable^[20]^. The parents were counseled about this and advised to have early prenatal ultrasonography and amniocentesis in the next pregnancies.

In this case, the patient’s family was unable to pursue genetic testing due to financial constraints, which is a common limitation in similar cases within resource-limited settings.

## Conclusion

Amelia is a rare condition that affects limb development. It can be isolated or associated with other anomalies. Diagnosing the etiology of amelia can be challenging. DUSS is an essential tool for the early diagnosis and management of similar cases.

## Data Availability

All data that support the findings of this study are included in this article. Further inquiries can be directed to the corresponding author.
